# Supplementary material for: Abiotic N2 reduction in submarine hydrothermal systems could quickly fertilize prebiotic oceans
Source: Nat Commun. 2025 Nov 28;16:10608. doi: 10.1038/s41467-025-65711-1 (PMC12663365; doi:10.1038/s41467-025-65711-1)
Supplement: Supplementary file 1 — Supplementary Information [file 41467_2025_65711_MOESM1_ESM.pdf]

Supplementary Information for

Abiotic N_2 reduction in submarine hydrothermal systems could quickly fertilize prebiotic oceans

Liheng Sun[1,2], Kan Li[2], Zhen Sun[1,3,4*], Yunying Zhang[1] and Long Li[2*]

[1]*State Key Laboratory of Tropical Oceanography, South China Sea Institute of Oceanology, Chinese Academy of Sciences, 510301 Guangzhou, China*

[2]*Department of Earth and Atmospheric Sciences, University of Alberta, Edmonton, Alberta, Canada T6G 2E3*

[3]*Key Laboratory of Marine Mineral Resources, Ministry of Natural Resources, Guangzhou Marine Geological Survey, China Geological Survey, Guangzhou 511458, China*

[4]*China-Pakistan Joint Research Center on Earth Sciences, CAS-HEC, 45320 Islamabad, Pakistan*

*Correspondence and requests for materials should be addressed to Z. Sun (email: sun_zhen2024@126.com) and L. Li (email: long4@ualberta.ca)

The Supplementary Information include:

Supplementary Discussion

1. Geological background, petrography and lithology.
2. Nitrogen sources of altered basalts at IODP Site U1502B.
3. Ammonium flux to support an early biosphere.

Supplementary Figures 1-8

Supplementary References

1. Geological background, petrography and lithology

Hole U1502B of the International Ocean Discovery Program (IODP) Expeditions 367 and 368 was drilled into the mid-ocean ridge basalt (MORB) on a prominent basement ridge (Ridge A; Supplementary Figure 1) at the seaward side of the continent-ocean transition of the northern South China Sea (SCS) margin. The oldest seafloor-spreading magnetic anomaly seaward of Site U1502 is Chron C11n (~ 30 Ma)[1,2], which runs parallel to Ridge A approximately 10 km to the south of the ridge.

The igneous basement recovered from Hole U1502B comprises of a 182 m-thick sequence of highly altered aphyric to plagioclase porphyritic basalts, which can be divided into two subunits based on flow morphology: an upper sequence of brecciated and fractured massive lavas (63 m; Figure 1) and a lower sequence of interbedded pillow flows with lobate and sheet lavas (119 m; Figure 1). Based on trace-element and REE distribution patterns (Supplementary Figure 2), U1502B basalts originated from E-MORB with slight enrichments in light REE (La_N/Sm_N of 0.84–1.33). The U1502B basalts have positive Pb and U anomaly compared to E-MORB, which may be attributed to hydrothermal fluid alteration (see more below).

All basalts have undergone variable degrees of hydrothermal alteration with multiple phases of veining and halos distributing throughout the core (Supplementary Figure 3). While the mineral assemblages of the basaltic units are similar, i.e., dominated by albite and quartz with minor amounts of augite, dolomite, chlorite, and pyrite (Supplementary Figure 3), the upper subunit contains more carbonate and clay (gray-brown alteration), whereas the lower subunit is dominated by sulfides, and chlorite (greenish gray-bluish gray alteration; Supplementary Figure 3). These indicate the upper subunit experienced additional low-temperature alterations (~50 °C)[2,4,5] than the lower subunit of the igneous basement.

Two types of veins have been observed in the igneous basement of Hole U1502B. Type 1 is white to green silicate veins (Supplementary Figure 3c, d), with albite, quartz, epidote, carbonates (dolomite/siderite/ankerite), chlorite, and minor pyrite. Based on quartz content, Type 1 veins can be further divided into silicate veins with high quartz contents and silicate veins with low quartz contents. Type 2 ones are white to brown carbonate veins (dolomite/calcite) with minor albite, quartz, chlorite and pyrite. The altered minerals of these silicate and dolomite veins are contemporaneous. A few calcite veins cut through or developed along the edges of silicate and dolomite veins, indicating that calcite veins were formed later than silicate and dolomite veins. The secondary mineral assemblages of altered basalts, silicate veins, and dolomite veins belong to the albite-epidote lower greenschist facies and thus indicate relatively high-temperature fluid-rock interaction at 200 to 300 °C[2,4,5].

2. Nitrogen sources of altered basalts

The significantly higher N contents of U1502B altered basalts in relative to fresh basalts from N-MORB or E-MORB sources[6-8] indicate assimilation of secondary NH_4^+, which could have occurred via hydrothermal alteration over a large temperature range[8,9]. Two sources could provide the secondary NH_4^+ to altered basalts. One is seawater-dominated shallow hydrothermal fluids, which contains NH_4^+ recycled from decomposition of organic matter, desorption of NH_4^+ from clays sediments[10], reduction of NO_3^- [11], which all have similar $\delta^{15}N$ values mainly falling in the range of +2‰ to +10‰ (average: ~ +6‰)[11,12]. The other is NH_4^+ synthesized within the hydrothermal system by abiotic reduction of mantle N_2, which can have $\delta^{15}N$ values varying from < –21‰ to –5‰, depending on the extent of the reduction reaction (see main text for discussion).

Accordingly, two-endmember mixing models were carried out for the addition of secondary NH_4^+ from each source into fresh basalts (Methods; Supplementary Figure 3b). The

$\delta^{15}N$ values of U1502B altered basalts vary from –7.6‰ to +0.2‰, which fall along a trend that cannot be explained by a single secondary source even applying the full $\delta^{15}N$ range of each of these sources mentioned above. Instead, the data trend points to involvement of both ^{15}N-enriched NH_4^+ from seawater-dominated shallow hydrothermal fluids and ^{15}N-depleted NH_4^+ from abiotic N_2 reduction in deep fluids.

The addition of NH_4^+ from these two sources could have occurred in different temporal and spatial scales. In the deep, relatively high-temperature hydrothermal systems, the NH_4^+ was mainly derived from the abiotic reduction process (with minor contribution from surface-derived NH_4^+; Figure 4 in main text). The NH_4^+ contents in these deep fluids may be relatively high but the bulk-rock alteration degrees are likely relatively low due to the low fluid/rock ratios as a result of the relatively low permeability in the massive/sheeted flow lavas. In contrast, in the shallow system near seafloor, the added NH_4^+ was mainly from seawater (with minor contribution of abiotic NH_4^+). The NH_4^+ contents in these seawater-dominated fluids are relatively low but the bulk-rock alteration degrees can be relatively high due to the large fluid/rock ratios as a result of the large permeability in the loosely piled pillow lava section. The N content of an altered basalt sample is a combined effect of all these factors (plus some more that have been introduced in main text), which are highly variable from location to location and thus difficult to predict.

3. Ammonium flux to support an early biosphere

It is interesting to assess whether an early biosphere could live off the estimated NH_4^+ flux from abiotic N_2 reduction. Considering that the most likely metabolic pathway of the microbial system living on ammonium in the reducing early oceans would be sulfate-reducing anaerobic ammonium oxidation, we can look into this question from the following two different approaches.

Firstly, in laboratory microbe culture experiments carried out in anaerobic environment with NH_4^+ and SO_4^{2-} in media, Mohammed Madani et al.[19] observed fast growth of biomass in their culture experiments at pH = 5 – 10 (optimal at 8) and T = 20 – 50 ℃ (optimal at 30 ℃), which well bracket the conditions of early-Earth oceans. The growth rates were up to 7.3×10^9 CFU/mL over 24 –72 hour experiments, which is equivalent to 3.65×10^{12} cells in the 500 ml flasks in their experiments. Although these experiments were carried out with a SO_4^{2-} concentration (~ 750 μM) slightly higher than the proposed SO_4^{2-} concentration in Archean oceans (2.5 – 200 μM)[20,21], these experimental results clearly demonstrated that the estimated sulfate and ammonium in the early oceans were capable of supporting a biosphere, likely with less biomass yielded in the experiments.

Secondly, from Gibbs energy point of view, we can provide a more quantitative assessment. We followed the calculation method of Schrum et al.[22] for the sulfate-reducing ammonium oxidation process:

$$8SO_4^{2-} + 3NH_4^+ \rightarrow 4N_2 + 3HS^- + 5H^+ + 12H_2O \quad (1)$$

$$\Delta G_{(T,P,S)} = \Delta G^0{}_{(T,P,S)} + RTln(\frac{\gamma_{N_2}{}^4[N_2]^4 \times \gamma_{HS^-}{}^3[HS^-]^3 \times \gamma_{H^+}{}^5[H^+]^5]}{\gamma_{NH_4^+}{}^8[NH_4{}^+]^8 \times \gamma_{SO_4^{2-}}{}^3[SO_4{}^{2-}]^3}) \quad (2)$$

in which ΔG is a function of pressure (P), temperature (T) and salinity (S); ΔG^0 is the standard reaction Gibbs energy; γ is activity coefficient (see details in Schrum et al.[22] and reference therein).

For a back-of-an-envelop calculation, we set the condition at fixed P of 1 bar and S of seawater salinity, but variable T of 15 – 70 ℃, pH of 6 – 8, $[SO_4^{2-}]$ from 2.5 – 200 μM[20,21]. The results in Supplementary Figure 8 show that, at pH = 6 – 8, sulfate-reducing ammonium oxidation can release energy when $[NH_4^+]$ accumulates to ~200 – 4000 μM under a $[SO_4^{2-}]$ of 200 μM or ~1000 – 15000 μM under a $[SO_4^{2-}]$ of 2.5 μM. Such $[NH_4^+]$ is possible in our calculations since

there was little sink for NH_4^+ in the prebiotic oceans. These energy yields enable to support a biosphere. However, due to the large uncertainty on the exact number of energy yield which is highly dependent on [NH_4^+] and pH, it is difficult to quantify the biomass at the moment.

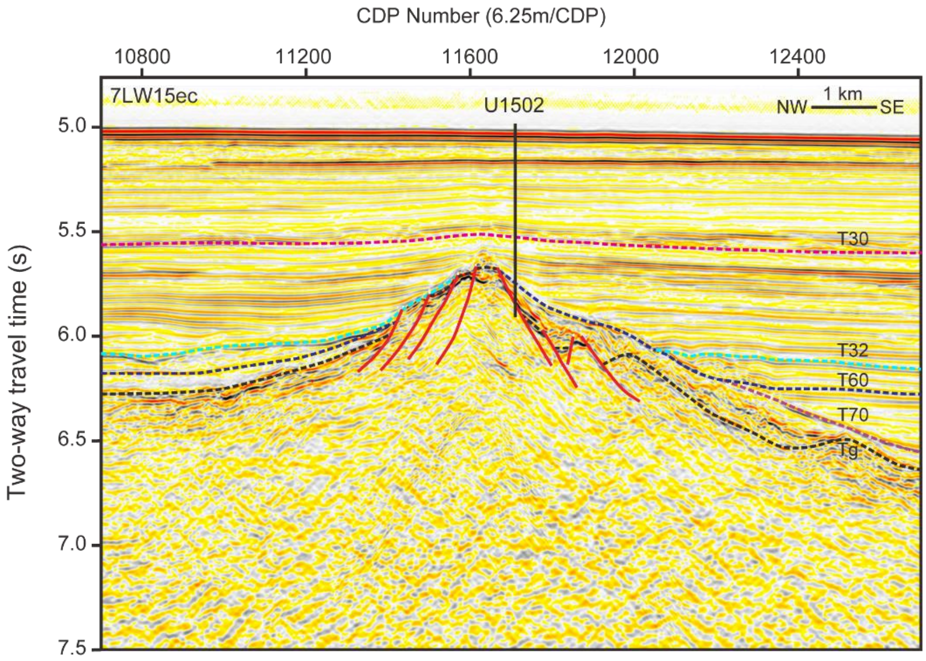

Supplementary Figure 1. Seismic section across Site U1502. Site U1502 was drilled into a prominent basement ridge (Ridge A) within the seaward end of the continent-ocean transition (modified after Larsen et al.[2]). Normal faults that dip (up to 50 – 60 °) both seaward and landward are well developed in the area around Ridge A. These normal faults do not extend upward into the Miocene sedimentary layer, and the sedimentary strata on two flanks show obvious onlap, indicating that the magmatic activity has stopped before the Miocene (T60; ~ 24 Ma).

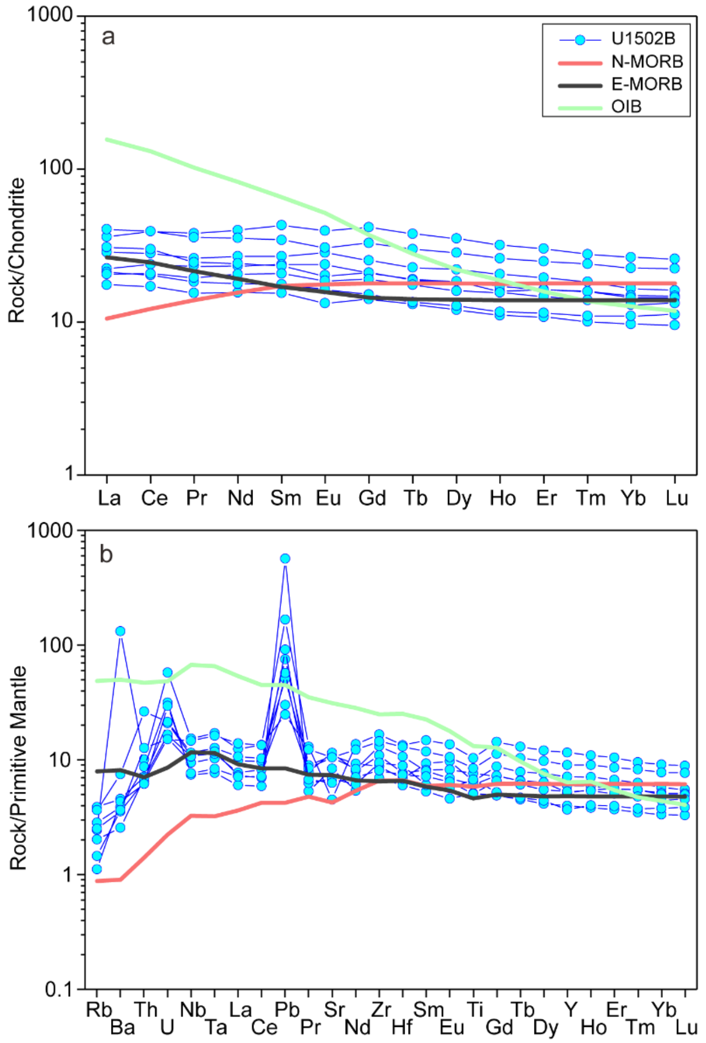

Supplementary Figure 2. Trace element patterns of Hole U1502B altered basalts. (a) Chondrite-normalized REE pattern; (b) Primitive mantle-normalized trace element pattern. The altered basalts are dominated by E-MORB patterns, but have positive Pb and U anomaly. Data of primitive mantle, chondrite, OIB, E-MORB, and N-MORB are from Sun and McDonough[3].

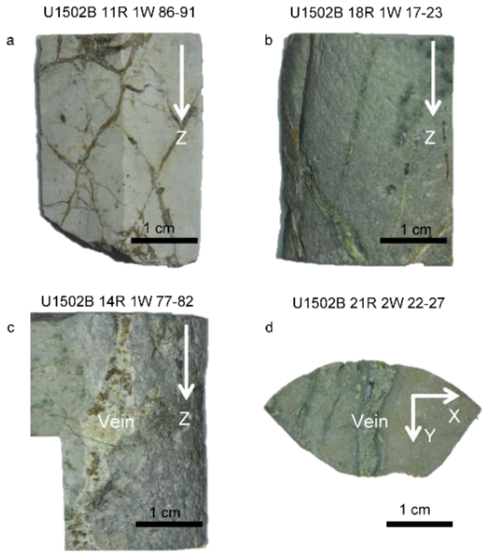

Supplementary Figure 3. Specimen photos of altered basalts and veins from IODP Site U1502B. (a) Altered basalt with gray-brown alteration; (b) altered basalt with greenish-bluish alteration; (c) and (d) veins in altered basalts. Sampling directions (Z: vertical; X and Y: horizontal) are also given.

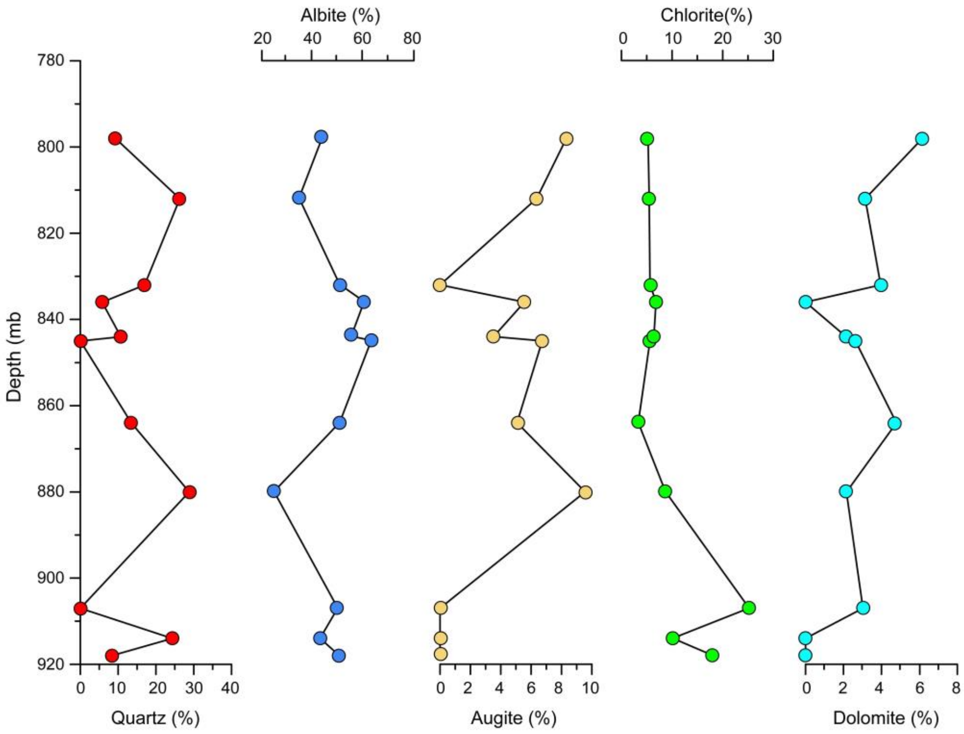

Supplementary Figure 4. Downhole variations of mineral contents in altered basalts from Hole U1502B.

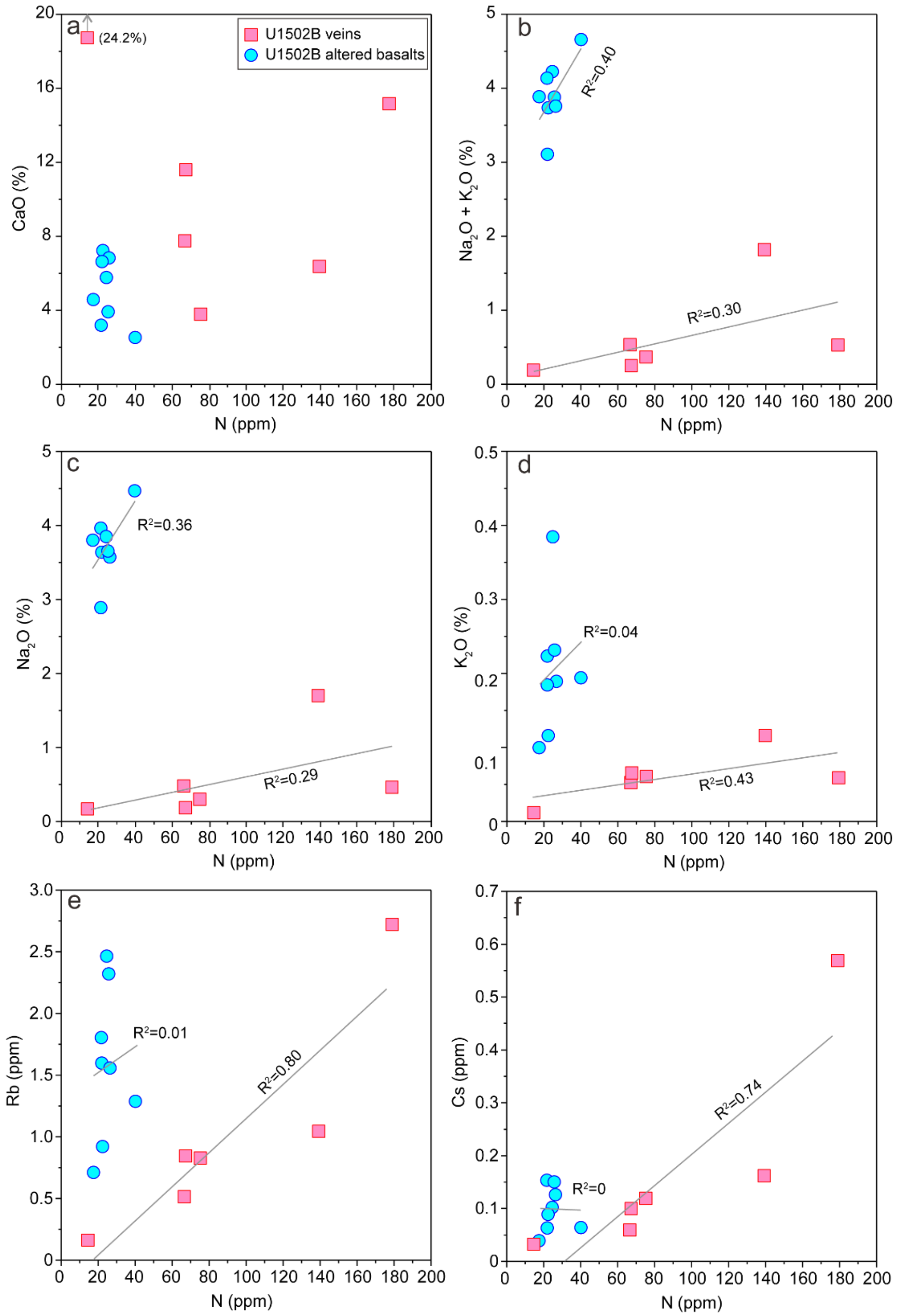

Supplementary Figure 5. Concentration comparison between nitrogen and other elements in U1502B altered basalts and veins. (a) N versus CaO; (b) N versus Na_2O + K_2O; (c) N versus Na_2O; (d) N versus K_2O; (e) N versus Rb; (f) N versus Cs.

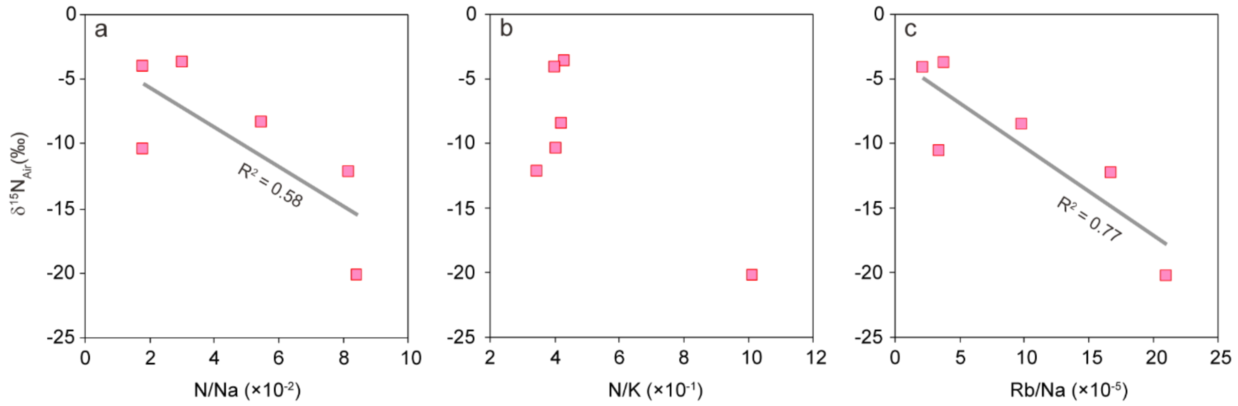

Supplementary Figure 6. Comparison of δ^{15}N value with elemental ratios of U1502B vein samples. (a) $\delta^{15}N$ versus N/Na; (b) $\delta^{15}N$ versus N/K; (c) $\delta^{15}N$ versus Rb/Na. All elemental ratios are molar ratios.

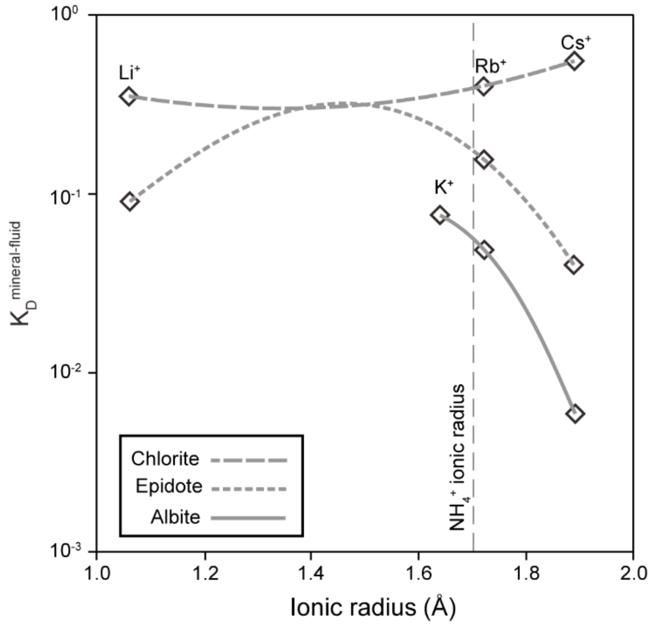

Supplementary Figure 7. Relationship between mineral-fluid partition coefficient and ionic radius for Li, K, Rb, and Cs in chlorite, epidote, and albite. The partition coefficient of each element in albite is the exchange coefficient ($K_D^{element-Na}$), while the partition coefficient of each element in chlorite and epidote is the simple partition coefficient (K_D)[13-16]. Following Blundy and Wood[17], the K_D values and radii of different elements in each mineral can be fitted by non-linear least-squares regression. The $K_D^{NH_4-Na}$ value for each mineral can be obtained by interpolating the NH_4^+ radius into the parabola relationship.

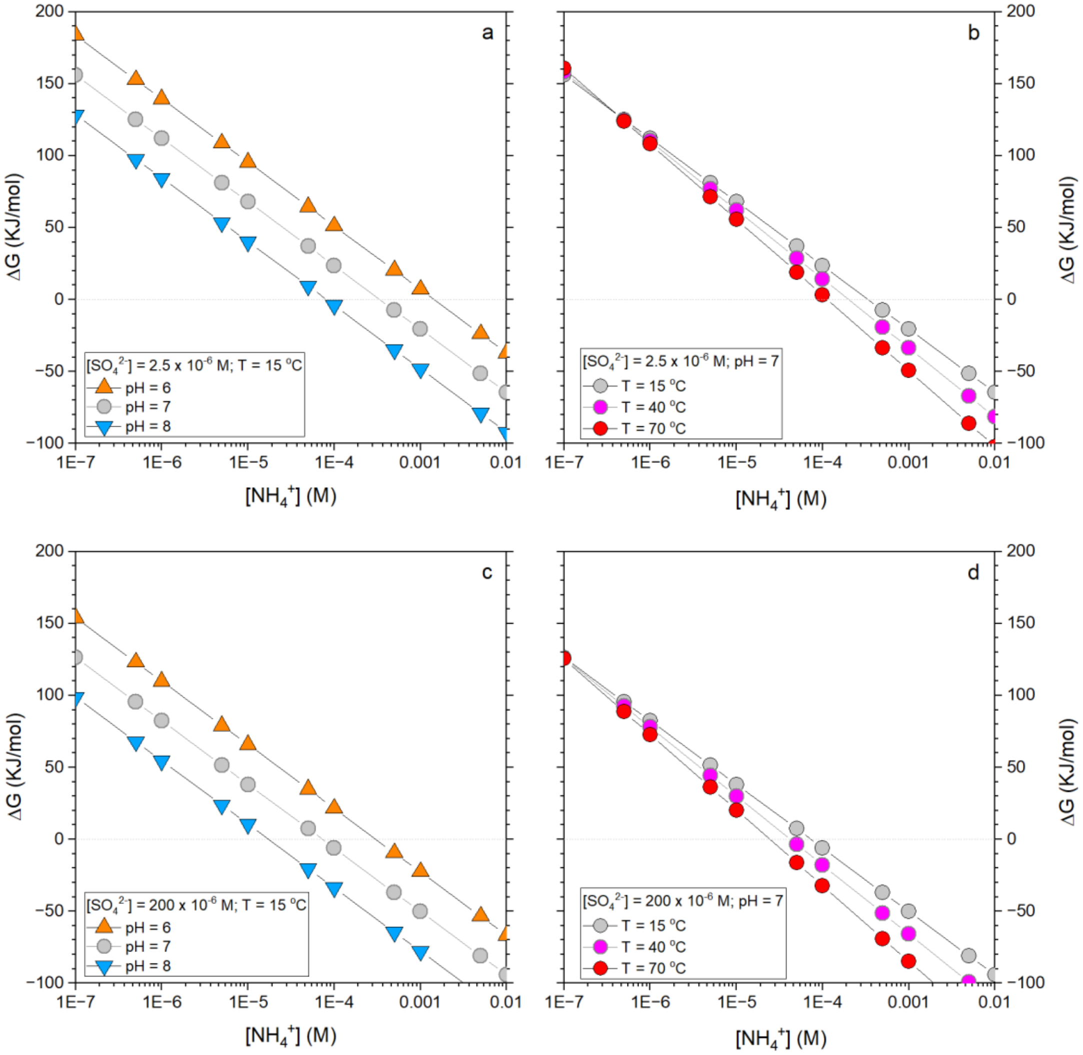

Supplementary Figure 8. Calculation of Gibbs energy for sulfate-reducing ammonium oxidation process. (a) Variable pH of 6 – 8 at fixed sulfate content of 2.5 μM and temperature of 15 °C; (b) variable temperature of 15 – 70 °C at fixed sulfate content of 2.5 μM and pH of 7; (c) variable pH of 6 – 8 at fixed sulfate content of 200 μM and temperature of 15 °C; (d) variable temperature of 15 – 70 °C at fixed sulfate content of 200 μM and pH of 7.

Supplementary References

1. Briais, A., Patriat, P. & Tapponnier, P. Updated interpretation of magnetic anomalies and seafloor spreading stages in the South China Sea: implications for the Tertiary tectonics of Southeast Asia. *J. Geophys. Res.* **98**, 6299-6328 (1993).
2. Larsen, H.C. et al. Site U1502. In Sun, Z. et al (ed.) *South China Sea Rifted Margin. Proceedings of the International Ocean Discovery Program, 367/368,* 1-77. College Station, TX (International Ocean Discovery Program) (2018).
3. Sun, S.-S. & McDonough, W. F. Chemical and isotopic systematics of oceanic basalts: Implications for mantle composition and processes. *Geol. Soc. London, Spec. Publ.* **42**, 313-345 (1989).
4. Chung, F.H. Quantitative interpretation of X-ray diffraction patterns of mixtures. II. Adiabatic principle of X-ray diffraction analysis of mixtures. *J. Appl. Crystallogr.* **7**, 526–531(1974).
5. Chen, L. X. et al. Seafloor hydrothermal circulation at a rifted margin of the South China Sea: Insights from basement epidote veins in IODP Hole U1502B. *Lithos* **444-445**, 107102 (2023).
6. Marty, B. & Humbert, F. Nitrogen and argon isotopes in oceanic basalts. *Earth Planet. Sci. Lett.* **152**, 101-112 (1997).
7. Bekaert, D.V., et al. A carbon, nitrogen, and multi-isotope study of basalt glasses near 14 °N on the Mid-Atlantic Ridge. Part B: Mantle source heterogeneities. *Geochim. Cosmochim. Acta* **369**, 179-195 (2024).
8. Li, L., Bebout, G. E. & Idleman, B. D. Nitrogen concentration and $\delta^{15}N$ of altered oceanic crust obtained on ODP Legs 129 and 185: insights into alteration-related nitrogen

enrichment and the nitrogen subduction budget. *Geochim. Cosmochim. Acta* **71**, 2344-2360 (2007).

9. Li, K. & Li, L. Nitrogen enrichment in the altered upper oceanic crust: A new perspective on constraining the global subducting nitrogen budget and implications for subduction-zone nitrogen recycling. *Earth Planet. Sci. Lett.* **602**, 117960 (2023).
10. Yu, A. J., Lin, X., Zhu, J., He, H., Li, L. Environmental effects on ammonium adsorption onto clay minerals: Experimental constraints and applications. *Appl. Clay Sci.* **246**, 107165.
11. Charoenpong, C. N. *The production and fate of nitrogen species in deep-sea hydrothermal environments*. PhD Thesis, Massachusetts Institute of Technology and Woods Hole Oceanographic Institution, pp 182 (2019).
12. Li, L., Zheng, Y.-F., Cartigny, P. & Li, J. Anomalous nitrogen isotopes in ultrahigh-pressure metamorphic rocks from the Sulu orogenic belt: Effect of abiotic nitrogen reduction during fluid–rock interaction. *Earth Planet. Sci. Lett.* **403**, 67-78 (2014).
13. Berger, G., Schott, J. & Guy, C. Behavior of Li, Rb and Cs during basalt glass and olivine dissolution and chlorite, smectite and zeolite precipitation from seawater: Experimental investigations and modelization between 50 °and 300 ℃. *Chemical Geology* **71**, 297-312 (1988).
14. Martin, L., Wood, B. J., Turner, S. & Rushmer, T. Experimental measurements of trace element partitioning between lawsonite, zoisite and fluid and their implication for the composition of arc magmas. *Journal of Petrology* **52**, 1049-1075 (2011).
15. Volfinger, M. & Robert, J. L. Structural control of the distribution of trace elements between silicates and hydrothermal solutions. *Geochim. Cosmochim. Acta* **44**, 1455-1461 (1980).

16. Shannon, R. D. Revised effective Ionic Radii and systematic studies of interatomic distances in halides and chalcogenides. *Act Crystallogr. A* **32**, 751-767 (1976).

17. Blundy, J. D. & Wood, B. Prediction of crystal-melt partition coefficients from elastic moduli. *Nature* **372**, 452-454 (1994).

18. Busigny, V., Cartigny, P., Philippot, P., Ader, M. & Javoy, M. Massive recycling of nitrogen and other fluid-mobile elements (K, Rb, Cs, H) in a cold slab environment: evidence from HP to UHP oceanic metasediments of the Schistes Lustrés nappe (western Alps, Europe). *Earth Planet. Sci. Lett.* **215**, 27-42 (2003).

19. Mohammed Madani, R. et al. Novel simultaneous removal of ammonium and sulfate by isolated Bacillus cereus strain from sewage treatment plant. *Water Air Soil Pollut.* **233**, 185 (2022).

20. Habicht, K.S., Gade, M., Thamdrup, B., Berg, P. & Canfield, D. E. Calibration of sulfate levels in the Archean ocean. *Science* **298**, 2372-2374 (2002).

21. Crowe, S. A. et al. Sulfate was a trace constituent of Archean seawater. *Science* **346**, 735-739 (2014).

22. Schrum, H. N., Spivack, A. J., Kastner, M. & D'Hondt, S. Sulfate-reducing ammonium oxidation: A thermodynamically feasible metabolic pathway in subseafloor sediment. *Geology* **37**, 939-942 (2009).
