# Supplementary material for: Abiotic N2 reduction in submarine hydrothermal systems could quickly fertilize prebiotic oceans
Source: Nat Commun. 2025 Nov 28;16:10608. doi: 10.1038/s41467-025-65711-1 (PMC12663365; doi:10.1038/s41467-025-65711-1)
Supplement: Supplementary file 3 — Description of Additional Supplementary Files [file 41467_2025_65711_MOESM3_ESM.pdf]

Description of Additional Supplementary Files

File name: Supplementary Data 1

Description: Mineral assemblages of altered basalts and veins from IODP Hole U1502B

File name: Supplementary Data 2

Description: Major and trace elements and nitrogen contents and $\delta^{15}N$ values of altered basalts and veins from IODP Hole U1502B

File name: Supplementary Data 3

Description: Partition coefficients (K_D) between minerals and hydrothermal fluids

File name: Supplementary Data 4

Description: Compilation of aqueous geochemical data for deep hydrothermal fluids
